# Network Structure of the Master Clock Is Important for Its Primary Function

**DOI:** 10.3389/fphys.2021.678391

**Published:** 2021-08-16

**Authors:** Changgui Gu, Jiahui Li, Jian Zhou, Huijie Yang, Jos Rohling

**Affiliations:** ^1^Business School, University of Shanghai for Science and Technology, Shanghai, China; ^2^Laboratory for Neurophysiology, Department of Cell and Chemical Biology, Leiden University Medical Center, Leiden, Netherlands

**Keywords:** SCN network, circadian rhythm, light-dark cycle, free running period, entrainment ability

## Abstract

A master clock located in the suprachiasmatic nucleus (SCN) regulates the circadian rhythm of physiological and behavioral activities in mammals. The SCN has two main functions in the regulation: an endogenous clock produces the endogenous rhythmic signal in body rhythms, and a calibrator synchronizes the body rhythms to the external light-dark cycle. These two functions have been determined to depend on either the dynamic behaviors of individual neurons or the whole SCN neuronal network. In this review, we first introduce possible network structures for the SCN, as revealed by time series analysis from real experimental data. It was found that the SCN network is heterogeneous and sparse, that is, the average shortest path length is very short, some nodes are hubs with large node degrees but most nodes have small node degrees, and the average node degree of the network is small. Secondly, the effects of the SCN network structure on the SCN function are reviewed based on mathematical models of the SCN network. It was found that robust rhythms with large amplitudes, a high synchronization between SCN neurons and a large entrainment ability exists mainly in small-world and scale-free type networks, but not other types. We conclude that the SCN most probably is an efficient small-world type or scale-free type network, which drives SCN function.

## Background

### Introduction of the Master Clock

There are many rhythmic processes present in our body, one of which is the so-called circadian rhythm, which helps our body to adjust to and anticipate daily changes in our environment. One of the most obvious circadian processes is our daily pattern of sleep and wakefulness, but we have daily patterns in eating, blood pressure, hormone regulation, and many more processes as well. All these processes are endogenously regulated by internal clocks. These clocks are synchronized by a master clock located in the hypothalamus of the brain, called the suprachiasmatic nucleus (SCN; Pittendrigh and Daan, 1976; Pittendrigh, 1993; Refinetti, 2006; Welsh et al., 2010). The main function of the SCN is its work as a calibrator: it entrains the body to the environmental 24-h cycle of light and darkness. But the SCN also acts as an endogenous clock. If an animal is removed from any environmental cycle and kept in either constant darkness or constant light conditions, the SCN still synchronizes the bodily rhythms with a period close to 24-h, the so-called free-running period. This free-running period differs between species (Ahlgren and Halberg, 1990; Czeisler et al., 1999; Daido, 2001). For example, the free running period is around 24.1 h for the Sudanian grass rat, 23.8 h for the southern flying squirrel, 22.9 h for deer mouse, and 24.2 h for human beings.

Interestingly, the SCN is also able to entrain the body rhythms to external cycles that deviate from the natural 24-h, which are so-called *T*-cycles (Campuzano et al., 1998; Abraham et al., 2010). There is a certain range in the cyclic periods that the SCN is able to entrain to, which is called the entrainment range. Within this range entrainment to the external *T*-cycle succeeds, outside this range entrainment is not achieved. This entrainment range differs from species to species. For the Sudanian grass rat the entrainment range is from 22.9 to 25.3 h, for the southern flying squirrel it ranges from 23.5 to 24.9 h, for deer mouse the range is from 22.5 to 25.1 h, and for humans the entrainment range is from 21.5 to 28.6 h (Refinetti, 2006).

The rhythms of the SCN originate from the individual neuronal oscillators. The individual neurons show oscillatory behavior in gene expression and electrical activity with intrinsic periods ranging from 22 to 28 h (Welsh et al., 1995; Honma et al., 2004). These neuronal oscillations arise from multiple interlocked feedback loops all involving Clock and Bmal1. A main transcriptional and translational feedback loop (TTFL) composed of Period and Cryptochrome genes and their protein products (Reppert and Weaver, 2001; Yan et al., 2008; Yi et al., 2021) is stabilized by two secondary TTFLs, the first involving REV-ERBα/β and ROR, and the second involving the PAR bZIP genes (Pilorz et al., 2020; Yi et al., 2021). For the purposes of this paper it is enough to understand that gene product mRNA produces protein products, which phosphorylate and subsequently inhibit the transcription of the genes, leading to less protein-inhibition and up-regulation of gene transcription, a continuous cycle with a period of approximately 24 h (Reppert and Weaver, 2001).

The robust rhythm of the SCN requires not only self-oscillatory neurons but also that the 20,000 neurons in the SCN synchronize their activity to create a sinusoidal rhythm that has a peak in the middle of the day and a trough in the middle of the night in gene expression (Yamaguchi et al., 2003). When the SCN neurons are not synchronized, i.e., the periods differ between neurons and the phase difference between neurons is not fixed, the overall rhythms of the SCN are lost even if the single neurons maintain their circadian rhythms in gene expression (Ohta et al., 2005). The existence of synchronization suggests that the SCN neurons communicate with each other and are coupled to each other in a network, where the neurons are regarded as the nodes and the coupling between neurons are the links.

The cellular coupling is achieved mainly through neurotransmitters and neuropeptides, such as γ -aminobutiric acid (GABA), vasoactive intestinal polypeptide (VIP), and arginine vasopressin (AVP), but also through electrical gap junctions, although only 26% of the neurons in the SCN are electrically coupled (Long et al., 2005), while all neurons in the SCN are reported to be GABAergic. GABA can either synchronize or desynchronize neurons, while VIP and AVP are thought to be mainly involved in the synchronization of groups of neurons in the SCN (Michel and Meijer, 2020; Pilorz et al., 2020). Also other cells, such as astrocytes, play a role in the synchronization in the network by suppressing the activity of the neurons during the night (Brancaccio et al., 2017). This makes the SCN a heterogeneous complex network of cells.

The rhythm of the molecular clock regulates the excitability of the neuron, but other neurons can also influence the molecular rhythm of a neuron by changing its activity phase (Meijer and Michel, 2014; Pilorz et al., 2020). However, it has also been shown that the molecular clock and the neuronal excitability can become dissociated (Buijink et al., 2020). Thus, the exact mechanisms of interaction between the molecular machinery of the neuron and the excitability is not yet identified.

Here, we will discuss the interesting question whether the clock and calibration functions of the SCN are determined by the properties of the individual neurons or the properties of the assembly of the neurons (Reppert and Weaver, 2002; Herzog, 2007; Welsh et al., 2010).

### Communities in the SCN

The SCN network can be grouped into functional and anatomical communities. The SCN is bilaterally situated above the optic chiasm, left and right of the third ventricle (Pickard and Turek, 1982; de la Iglesia et al., 2000). The communication or coupling mechanism between the right SCN and left SCN is still not fully understood. Normally, the two SCN parts are assumed to function in unison. However, in certain very specific circumstances these two parts can become desynchronized and the left and right SCN start to oscillate in anti-phase, meaning that a phase difference of the two components stabilizes to 12 h (or π) (de la Iglesia et al., 2000; Ohta et al., 2005). This phenomenon is called splitting and can even be observed in the behavior of the animal, where the animal shows two bouts of behavioral activity in each 24-h cycle. The split SCN activity is shown in physiological function, and it shows that both components have an equal contribution to the behavior of the animal, indicating a symmetrical coupling between both components.

**FIGURE 1:**
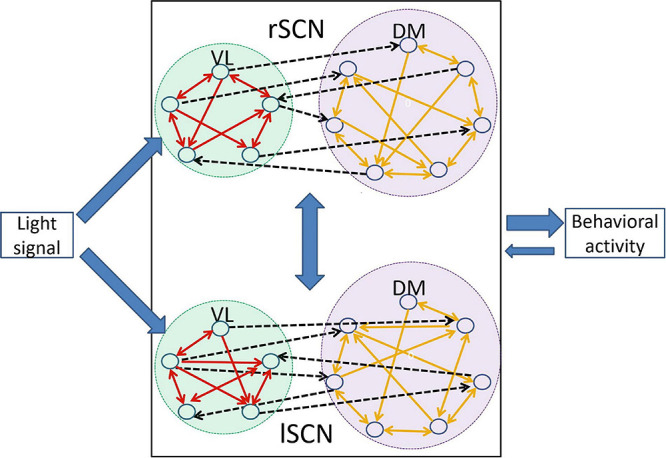
Schematic drawing of the SCN organization. The left and right SCN are equal and consist of a VL and DM part. The VL receives the light information and relays the light information to the DM. The solid arrows represent directed links between neurons within the VL or the DM, and the dashed black arrows represent the directed links between neurons in the VL and DM. Note that there are more directed links from the VL to the DM than vice versa. The SCN regulates the circadian rhythms of behavioral activity which also feeds back to the SCN.

The structure of the left and right SCN are comparable, and are generally divided into a ventral core region and a dorsal shell region. Approximately 25% of the neurons are sensitive to light (Meijer et al., 1986), and the majority of these neurons is believed to reside in the ventral part of the SCN (Lee et al., 2003), although retinal ganglion cell projections are found also in the dorsal part of the SCN (Fernandez et al., 2016), and light activates c-FOS throughout the SCN (Castel et al., 1997). Although light sensitivity of this SCN network is quite complex, in this review a light-sensitive group and a light-insensitive group will be distinguished, and as the majority of light-sensitive neurons resides in the ventral region of the SCN, the light-sensitive group will be called “VL,” and will be associated with the ventral cluster, while the light-insensitive group will be called “DM” and associated to the dorsal cluster. Thus, the light-receptive VL contains approximately 25% of the neurons in the SCN and the light-insensitive DM possesses the remaining 75% of the neurons (Meijer et al., 1986; Meijer and Schwartz, 2003; VanderLeest et al., 2009; Rohling et al., 2011). The VL receives photic information from the retinohypothalamic tract (RHT), and relays the light information to the DM which is insensitive to the light-information. Unlike the symmetrical coupling between the left and the right SCN, the coupling between the VL and the DM is asymmetrical, i.e., the VL dominates the DM. This asymmetry can be explained by the distribution of neurotransmitters. The VL neurons mainly produce VIP which is absorbed by both VL and DM neurons, although the function of VIP in the DM neurons is not fully understood (Evans, 2016), while the DM neurons mostly express AVP to which the VL is insensitive (Albus et al., 2005; Aton et al., 2005; Silver and Schwartz, 2005). The communication between VL and DM mostly relies on GABA (Albus et al., 2005). As such, the SCN is a heterogeneous network composed of distinct communities. Figure 1 shows a schematic diagram of the SCN network and the input/output of SCN.

### The Rhythmic Behaviors of the SCN Under Typical Conditions

The community structure of the SCN network can be examined when animals in experimental conditions are exposed to several light conditions, such as constant darkness, constant light, different *T* cycles, jet-lag protocols, and seasonal changes in day-length resulting in short and long photoperiod. In experiments where rats were exposed to constant light conditions, rats could either keep their circadian rhythm in behavior, or their rhythm could be split into two bouts of activity in each 24-h day, or they could lose their circadian rhythm altogether (Pickard and Turek, 1982; de la Iglesia et al., 2000). It appeared that the neurons in the SCN did remain rhythmic in the rats that lost their rhythm, but the phase-synchronization between the neurons was lost. For the rats that remained rhythmic, this synchronization did remain (Ohta et al., 2005). Therefore, the synchronization in phase of activity of the neurons plays a key role in the rhythmic output of the SCN.

In experiments where rats were exposed to a *T* cycle with a period of *T* = 22 h, an interesting phenomenon emerged called “dissociation” (de la Iglesia et al., 2004). Two periodic components were observed in the rhythms of the behavioral activity, one of which showed a period equal to *T* = 22 h and the other showed a period close to the free running period being approximately 24 h (de la Iglesia et al., 2004). Further studies revealed that the 22 h component and 24 h component were produced by the VL and the DM, respectively (Schwartz et al., 2009). Furthermore, in a jet-lag protocol, where animals got a 6-h delay of the light-dark cycle, for example, the SCN requires several days to recover its robust circadian rhythm. In detail, the VL abruptly shift to the phase (time) of the new cycle immediately, while the DM stays in phase with the previous light-dark cycle, and only gradually shifts toward the phase of the new cycle in about 6 days (Albus et al., 2005; Rohling et al., 2011). These two results support that the VL is sensitive to the light information and the DM is not, and the coupling strength from the VL to the DM is larger than in the other direction.

It has also been found that the SCN network rhythms are affected by the photoperiod length. The amplitude of SCN rhythms differs between the short photoperiod, e.g., 8 h:16 h light:dark cycle (representing winter days: short days and long nights) and the long photoperiod, e.g., 16 h:8 h light:dark cycle (representing summer days) (VanderLeest et al., 2007; Meijer et al., 2010; Gu et al., 2016). This difference results from the degree of synchronization of the activity phase of the neurons and not from the amplitude of the single neurons. In the long photoperiod, the phase-synchronization is less strict than in the short photoperiod, resulting in an overall decrease in amplitude of the SCN network rhythm in long photo-periods compared to short photo-periods (VanderLeest et al., 2007; Meijer et al., 2010; Buijink et al., 2016; Gu et al., 2016).

In addition to the different light conditions, drugs, such as tetrodotoxin (TTX), can also be applied to the SCN. TTX is known to completely abolish intercellular coupling between neurons. After the application of the TTX to a brain slice, the synchronization between neurons is deteriorated since the coupling through the neurotransmitters is blocked, leading also to a reduction of the amplitudes of single neurons (Yamaguchi et al., 2003; Abel et al., 2017; Zhou et al., 2020). Removal of the TTX leads to the restoration of the synchronization and the amplitudes. This result suggests that there is a relationship between the synchronization (or coupling) and the amplitudes of the single neurons.

### The Organization of the Article

The experiments mentioned above showed that the SCN function depends on the collective behavior of the SCN neurons, and the synchronization or coupling between the neurons plays a key role in the rhythm of the SCN. The synchronization of activity between neurons means that these neurons communicate about their activity patterns, and thus a network of connections is present between the neurons. A network is generally described as a set of nodes (the neurons) and a set of links (the interconnections between the neurons, also often called *edges* in network literature) (Abel et al., 2017). The SCN network is heterogeneous in both the properties of the nodes themselves and the structure of the network connections. The heterogeneity of nodal properties lies in the difference of the sensitivity to light between VL and DM neurons, and in the intrinsic neuronal periods as well as the intrinsic neuronal amplitudes of the different SCN neurons (Gu and Yang, 2017; Gu et al., 2019c; Zhu et al., 2020). The heterogeneity of the network structure includes the asymmetrical coupling between the VL and the DM and the connectivity within these communities. Are all neurons connected to all other neurons, in the communities and between communities? It is very costly in energy expenditure in the brain to maintain such a network, so this is not very probable. But how are the neurons connected? What is the network structure of the SCN, within and between communities? And how does this network structure affect the functionality of the SCN?

In the following sections, we will explore the SCN network structure and its effects on the SCN functions. The remainder of this review is organized as follows. In section “Network Structure Explored by Time-Series Analysis,” we will summarize how to identify the network structure using time series analysis methods. Then, in section “Circadian Clock Model,” we introduce a Goodwin-type model which describes a single neuronal oscillator (Gonze et al., 2005), and expand this model with SCN network connectivity. Based on the mathematical model, section “Collective Behaviors of the Neurons Affected by the SCN Network Structure” will present the effects of the network structure on SCN function. Finally, the conclusions and discussions are presented in section “Discussion and Conclusion.”

## Network Structure Explored by Time-Series Analysis

### Introduction to Complex Networks

**FIGURE 2:**
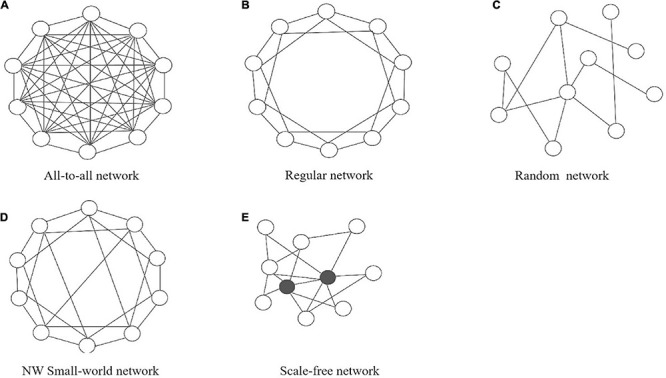
Schematic drawing of the five typical network types: **(A)** all-to-all network, **(B)** regular network, **(C)** random network, **(D)** NW small-world network, and **(E)** scale-free network.

Several typical types of the network structures (or topologies) have been widely studied in a lot of fields, including all-to-all connectivity networks, regularly connected networks, Newman-Watts (NW) small-world networks and scale-free networks (Vasalou et al., 2009; Hafner et al., 2012; Gu and Yang, 2016; Gu et al., 2019a). For simplicity, we will first consider undirected networks, where the connections between nodes always go both ways. In an all-to-all network, every node is connected to every other node. Therefore, there are $\frac{N^{2}}{2}$ links where *N* is equal to the number of nodes. In a regular network, two nodes are connected if their physical distance is smaller than some critical value, so it is also called nearest-neighbor network. An NW small-world network starts as a regular network, but additional long-range connections are randomly added between nodes according to a certain probability. Finally, a scale-free network is characterized by large numbers of nodes that have very few connections to other nodes, while there are just a few nodes, called hub-nodes, that have gathered many connections to many other nodes. Figure 2 shows a schematic diagram of several network types.

Network topologies have a number of classical statistical properties, such as the distribution of node degrees, average shortest path lengths and cluster coefficient (Albert and Barabasi, 2002). The degree of a node is defined as the number of links going to or from this node. The node degree represents the importance of the node to some extent. If the degree of a node is evidently larger than most nodes, it is called a hub. The shortest path length is the minimal number of steps through the network from a node to reach any other node in the network. The average shortest path length of the network as a whole is the average over all shortest path lengths from all individual nodes. The cluster coefficient for a node represents the possibility for two connected nodes of the node to also have a link. If the cluster coefficient for a node is 0, no link exists between any of the nodes connected to this node. If the cluster coefficient for a node is 1, a link exists between every two nodes this node is connected to. The cluster coefficient for the whole network is the mean of the cluster coefficients for all nodes. Distinct network topologies possess different properties (see Table 1). For example, for a NW small-world network the average shortest path length is short and the cluster coefficient is large, while in a scale-free network the node degrees satisfy a power-law distribution and the average shortest path length is short. In a regular network both average shortest path length and cluster coefficient are large (Albert and Barabasi, 2002).

**TABLE 1:** Network properties for typical network types.

**Network types**	**Network properties**		
	**Degree distribution**	**Average path length**	**Cluster coefficient**
All-to-all network (Albert and Barabasi, 2002; Newman, 2003)	*k* = *N*−1	*L* = 1	*C* = 1
Regular network (Albert and Barabasi, 2002; Newman, 2003)	*k* = *K*	$L\approx \frac{N}{2K}$	$C=\frac{3\left(K-2\right)}{4\left(K-1\right)}$
Random network (Bollobás, 2001)	$P\left(k\right)=C_{N}^{k}p^{k}{\left(1-p\right)}^{N-k}$	$L\propto \frac{\ln N}{\ln p\left(N-1\right)}$	*C* = *p*
NW Small-world network (Newman, 2002)	$\begin{array}{c} P\left(k\right)=C_{N}^{k-K}{\left(\frac{Kp}{N}\right)}^{k}{\left(1-\frac{Kp}{N}\right)}^{N-k+K}\\ k\geq K \end{array}$	$L=\frac{N}{2db}F\left(pbN^{d}\right),N=\xi$	$C=\frac{3\left(K-2\right)}{4\left(K-1\right)+4Kp\left(p+2\right)}$
Scale-free network (Dorogovtsev et al., 2000; Cohen and Havlin, 2003; Fronczak et al., 2003)	$P\left(k\right)=\frac{2m\left(m+1\right)}{k\left(k+1\right)\left(k+2\right)}$	$L\propto \frac{\log N}{\log\log N}$	$C=\frac{m^{2}{\left(m+1\right)}^{2}}{4\left(m-1\right)}\left[\ln\left(\frac{m+1}{m}\right)-\frac{1}{m+1}\right]\frac{{\left[\ln t\right]}^{2}}{t}$

**N* is the number of nodes, *k* is the degree of the node, *K* is the degree of the node in random network and is a fixed value in NW small-world network, *p* is the probability of two nodes connected, ξ is Characteristic length unit, *b* is the number of network edges, *d* is degree of separation, and $\xi =\left\{ \begin{array}{c} \frac{1}{pd},d=1\\ \frac{1}{pbd^{\frac{1}{d}}},d>2 \end{array}\right.$, *F* is an universal computing function and $F\left(x\right)=\left\{ \begin{array}{c} \frac{1}{4},x<1\\ \frac{\log2x}{4x},x\geq 1 \end{array}\right.$, *m* is the number of existing nodes and *t* is the step size in building a scale-free network.*

For the discovery of the network topology of the SCN, data is needed preferably from many individual neurons in the SCN. The dynamic behaviors of single neurons can be tracked for over one week by bioluminescence recordings in brain slices. This method can detect the dynamic behavior of hundreds of neurons simultaneously (Yamaguchi et al., 2003; Abel et al., 2017). The recording data for one neuron is a time series covering up to one week of data, where the sampling interval is one hour. Using these time series, the existence of link between two neurons can be determined by calculating correlations between the time traces of the different neurons: the more similar the time series of two neurons are, the higher the probability that these neurons are somehow functionally connected (Abel et al., 2017). There are multiple correlation techniques that can be used to estimate a connectivity value between each pair of neurons. From this information, the network of the SCN can be determined.

### Community Detection of the VL and the DM

**FIGURE 3:**
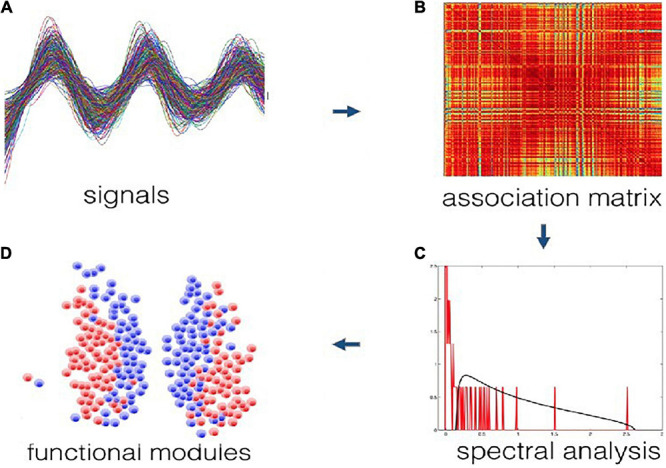
Illustration of the procedure of functional module identification from time series data **(A)** to data clusters **(D)**. First a correlation matrix is constructed based on the data **(B)**. The empirical correlation spectrum is then compared against a null model specifically tailored for correlation matrices **(C)**. This produces a filtered correlation matrix that is subsequently searched for modules, represented by the peaks of the spectral analysis. These modules are guaranteed to be statistically significant, noise-free, overall positively correlated internally and overall negatively correlated across **(D)**.

Using an impartial community detection method on bioluminescence data, we found two main communities in the SCN. Briefly, a cross-correlation matrix was constructed based on raw time series of gene expression from individual neurons in the SCN. Using random matrix theory local (neuron-specific) noise and global (SCN-wide) dependencies were filtered from the correlation matrix (Macmahon and Garlaschelli, 2015). More specifically, the method uses a modified Wishart ensemble of random correlation matrices, which is constructed using a similar common trend and expected noise level as the empirical time series, but under the null hypothesis that no modular organization is present. If the empirical and null correlation matrices are compared, the functional modules are revealed that are present in the data and absent in the model (Almog et al., 2019). The method is purely data-driven, and does not have any knowledge on locations of the neurons that are recorded. It also does not use arbitrary thresholds to identify communities. It guarantees to find a partition of the data into communities that are positively correlated internally and negatively correlated between the communities (Almog et al., 2019). Figure 3 shows the illustration of the procedure of functional module identification for this method.

We gathered PER2::LUC gene expression data from single neurons in acutely prepared SCN slices from young and aged mice. Both age groups were divided in two different groups, where one group was subjected to short photoperiods (mimicking winter days) and the other group was subjected to long photoperiods. Brain slices were taken after the animal was entrained to the photoperiod and the SCN was prepared for the bioluminescence recording of expression of the Per2 protein. Each hour a picture was taken for one week. ROI’s reflecting single neurons were identified in the stack of pictures and for each identified neuron a time trace was extracted from the pictures, resulting in a time series of bioluminescence values each hour for approximately 200 neurons for each experiment (Buijink et al., 2016, 2020).

The community detection method used the time series of all the neurons in each experiment, and it systematically uncovered two main communities in all experiments. It was shown that in short photoperiods, the positive internal correlations and the negative across correlations were higher than in long photoperiods, confirming the higher connection strengths expected in short photoperiods (Almog et al., 2019). The communities that were found not always exactly overlapped with the anatomical VL/DM distinction, but they are comparable to the regions described in other literature (Buijink et al., 2016, 2020). Thus, we believe that we found solid evidence for a modular system in the SCN consisting of two main communities, where one could be light-sensitive and the other is not sensitive to light.

### Properties in the Undirected SCN Network

It has now been established that the network of the SCN is modular, and contains different communities of neurons. However, it is also important to investigate the actual functional connections between these neurons: which neurons are connected and which are not? In Abel et al. (2017), an undirected network is identified, which contains about 400 neuronal nodes per slice. For each pair of neurons a correlation value was calculated based on a mutual information method. An undirected link between two neurons exists if the correlation value between two neurons is larger than a threshold.

In each of the experimental slices that were investigated, the SCN network satisfied small-world characteristics (Abel et al., 2017). An exponential distribution of node degrees was found, as well as a small average shortest path length (around 4) and a large average clustering coefficient (around 0.35). In accordance with the experiment results, the authors found that there were also hubs in this small-world network, and these are preferentially located in the central SCN, with sparsely connected shell-regions surrounding the core. This indicates that the network might also contain some scale-free properties. The shortcoming of this method is that the links are undirected, and therefore does not take the asymmetrical coupling between the VL and the DM, which suggests a directed connection, into account.

### Properties in Directed SCN Network

In order to explore the directed network structure of the SCN, two articles performed an analysis on the data from Ref. (Abel et al., 2017) using the method of information theory based technique (McBride and Petzold, 2018) and the method of the transfer entropy (Gu et al., 2019a). It was found that the incoming degree is similar for most SCN neurons but the outgoing degree varies between SCN neurons and the nodes with large outgoing degree are located in the core (McBride and Petzold, 2018). We found that the SCN network for each slice partially is a scale-free network (Gu et al., 2019a). A power-law distribution of the node degrees was found, as well as a short averaged path length (around 2.5). However, we also found a small clustering coefficient, which does not comply to the small-world characteristics.

**FIGURE 4:**
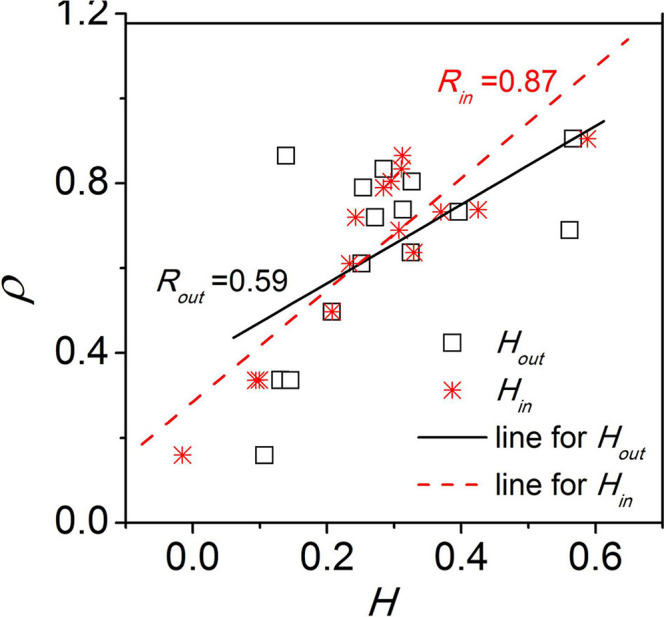
The relationship between the synchronization degree (*ρ*) and the disassortativity degree (*H*). *H*in** and *H*out** represents the disassortativity degree calculated based on the incoming degree and outgoing degree of nodes, respectively. This figure is modified from Figure 4 in Gu et al. (2016).

Additionally, we examined whether the network structure is disassortative or assortative (Gu et al., 2019a). In the disassortative network, the nodes preferably connect to other nodes with a different node degree, while in the assortative network, the nodes preferably connect to nodes with a similar node degree. The SCN network was found to be disassortative with respect to both the incoming degree and the outgoing degree of the nodes. The disassortative network is characterized by a disassortativity coefficient which signifies the heterogeneity in degree correlations between nodes in a network. We found that the synchronization between neurons are correlated to the disassortativity coefficient (Figure 4) as well as to the scaling exponent of the power-law distribution of node degrees. Therefore, the properties of the network structure affect the collective behavior of the SCN and thereby affect the function of the SCN. Unfortunately, limited to the current experimental technology, it is impossible to infer the exact network structure of the SCN. Accordingly, a networked model is built to examine the effects of different possible SCN network structures on SCN function.

## Circadian Clock Model

### Goodwin Model for One Neuron

The Goodwin model has been widely used to model circadian rhythms of mammals (Ruoff and Rensing, 1996; Leloup et al., 1999; Gonze et al., 2005; Li et al., 2006; Locke et al., 2008; Gu et al., 2009; Ullner et al., 2009; Gu et al., 2011, 2012, Xu et al., 2012). In this model, a single SCN neuron is represented by three variables, i.e. one for clock gene mRNA, one variable for clock protein, and one for a transcriptional inhibitor, and can be written as:

(1) $$\begin{array}{c} \dot{x}=\alpha_{1}\frac{k_{1}^{n}}{k_{1}^{n}+z^{n}}-\alpha_{2}\frac{x}{k_{2}+x}+L,\\ \dot{y}=k_{3}x-\alpha_{4}\frac{y}{k_{4}+y},\\ \dot{z}=k_{5}y-\alpha_{6}\frac{z}{k_{6}+z}, \end{array}$$

where the clock-gene mRNA concentration *x*, the clock-protein concentration*y*, and a transcriptional inhibitor concentration *z* constitute a negative feedback loop for the SCN neuron. *L* denotes the sensitivity to light information from the retino-hypothalamic tract. If *L* is equal to 0, the neuronal oscillator does not receive any light information; if *L* is a constant value that is larger than 0, the neuronal oscillator receives light information constantly; and if the alternation of *L* is periodic, i.e., *L* = *K*_*f*_*mod*(*t*,*T*) < *T*/2 and *L* = 0*mod*(*t*,*T*)≥*T*/2, the model mimics the situation of an external light-dark cycle with period *T*.

### Networked Goodwin Model

The networked Goodwin model describes how multiple Goodwin oscillators interact with each other, and is written as:

(2) $$\begin{array}{c} \overset{.}{\underset{i}{x}}=\alpha_{1}\frac{k_{1}^{n}}{k_{1}^{n}+z_{i}^{n}}-\alpha_{2}\frac{x_{i}}{k_{2}+x_{i}}+\alpha_{c}\frac{gF_{i}}{k_{c}+gF_{i}}+L_{i},\\ \overset{.}{\underset{i}{y}}=k_{3}x_{i}-\alpha_{4}\frac{y_{i}}{k_{4}+y_{i}},\\ \overset{.}{\underset{i}{z}}=k_{5}y_{i}-\alpha_{6}\frac{z_{i}}{k_{6}+z_{i}},\\ \overset{.}{\underset{i}{V}}=k_{7}x_{i}-\alpha_{8}\frac{V_{i}}{k_{8}+V_{i}},i=1,2,\cdots ,N\\ F_{i}=\frac{1}{\sum_{j=1}^{N}e_{ji}}\sum_{j=1}^{N}V_{i}e_{ji}, \end{array}$$

where subscript *i* denotes *i*_*th*_ clock neuron, and *N* is the total number of SCN neurons. *e*_*ji*_,*i*,*j* = 1, 2,⋯,*N* is an element of the adjacency matrix *A*_*N*×*N*_ which describes the network topology. If *e*_*ji*_ = 1, there is a directed link from *j* to *i*; and if *e*_*ji*_ = 0, there is no directed link from *j* to *i*. Note that *e*_*ji*_ is not necessarily equal to *e*_*ij*_. In Vasalou et al. (2009), the self-link is not allowed, i.e., the node is not linked to itself; while in Bernard et al. (2007), the node can be linked to itself through autocrine activation. $\sum_{j=1}^{N}e_{ji}$ is equal to the number of *i* incoming neighbors, i.e., the incoming degree for node *i*. *F*_*i*_ performs as a local mean-field which is the mean value of the transmitters (e.g., VIP) produced from *i*’s incoming neighbors. Typically, the model of all-to-all network is reduced to the model of global mean field, where each element *e*_*ji*_,*i*,*j* = 1, 2,⋯,*N* is equal to 1 (Gu et al., 2016). *g* is regarded as a coupling strength to represent the sensitivity of the neuron *i* to the local mean field *F*_*i*_, which is assumed to be identical for each neuron. We will examine the effects of the network structures described by *e*_*ji*_ on the collective behaviors of the neurons based on the mathematical models in section “Collective Behaviors of the Neurons Affected by the SCN Network Structure.”

The light-sensitive term *L*_*i*_ differs between neurons. Only the SCN neurons situated in the VL are sensitive to light information, while the neurons in DM are not. Thus, the term *L*_*i*_ can be represented by:

(3) $$\begin{array}{c} L_{i}=K_{f},mod\left(t,T\right)<T/2\;i=1,2,\cdots ,pN\\ L_{i}=0,mod\left(t,T\right)\geq T/2\;i=1,2,\cdots ,pN\\ L_{i}=0,i=pN+1,pN+2,\cdots ,N \end{array}$$

Where *p* is the ratio of neurons being sensitive to light information, and *pN* is the number of SCN neurons sensitive to light information.*K*_*f*_ is the light intensity, and *T* is the period of external *T*-cycle.

## Collective Behaviors of the Neurons Affected by the SCN Network Structure

Achievements from the networked SCN model have three aspects. First, a possible network structure can be suggested for the SCN after several potential types of network models are compared. Second, the effects of the SCN network structure on the collective behaviors of the neurons can be examined based on models, while this examination seems unfeasible with current experimental technologies. Third, the models can provide an explanation for experimental phenomena or results.

In this section, we will review the effects of network structures on the collective behaviors, e.g., the synchronization between the neurons, free running periods and entrainment ability under typical light conditions. Several types of networks for the SCN are compared in this review, including an all-to-all network, a regular network, a random network, a small-world network, and a scale-free network. Additionally, we review the communities divided by the VL and the DM, and communities divided by the right and the left SCN.

### All-to-all Network

The easiest way to introduce coupling between neurons in a neuronal network model of the SCN is through a global mean-field of the neurotransmitter concentration (Gonze et al., 2005; Locke et al., 2008; Gu et al., 2009). With a global mean-field coupling, the SCN is modeled as an all-to-all network where the link strengths between every neuron-pair are identical, i.e., 1 for simplicity. These modeling studies found that the coupling strength between nonidentical neurons plays a key role in the collective behavior of the SCN. With the increase of the coupling strength, the phase-synchronization between the neurons improves and the amplitudes of the neurons increases. As a result, the amplitude of the SCN rhythm increases.

Additional to the increasing amplitudes, the rigidity of the SCN is also related to the coupling strength, that is, the rigidity increases with the increase of coupling strength. Due to the increase of both the amplitude and rigidity, a negative relationship exists between the entrainment range of the SCN and the coupling strength, provided all neurons are sensitive to the external stimulus (e.g., entrainment due to temperature pulses) (Abraham et al., 2010). Interestingly, a different relationship was observed between the entrainment range of the SCN and the coupling strength if only a portion of neurons are sensitive to the external stimulus (e.g., the VL community in the light-dark cycle). In this case, the relationship is parabolic-like, in other words, with the increase of the coupling strength, the entrainment range increases if the coupling strength is smaller than a critical value, but decreases if the coupling strength is larger than this critical value (Gu et al., 2014). Although it is known that there is not all-to-all coupling in the SCN, these results are a strong indication that the coupling strength between the neurons do have an effect on the collective behavior of the SCN neurons.

Closer to reality, the link strengths may depend on the physical distance between each nodes (To et al., 2007). If two nodes are close, the strength of the coupling is larger, and the further away the nodes are from each other, the weaker the coupling strength becomes. Particularly, the nodes are located on the grids in the plane. If the nodes are adjacent on the grid, the strength is largest, i.e., 1, and if the nodes are connected diagonally, the strength is second largest, i.e., $\frac{1}{\sqrt{2}}$. Therefore, every node is influenced more by the nodes that are in close proximity, and less by nodes that are further away. Furthermore, they divided the cells in two groups, one of which was light-sensitive, while the other group was not. The authors found that both the coupling and the heterogeneity of the population affects the synchronization degree, and they suggested that both stochastic (cell-to-cell variability) and deterministic (network architecture) phenomena are important to understand the synchronization in phase between neuronal activity in the SCN. Additionally, it was found that there exists an asymmetry between the process of synchronization and desynchronization when the SCN is exposed to the constant light. Desynchronization was a slow process, extending over 3–6 days as oscillators slowly drifted out of phase, while (re-)synchronization was quite rapid, achieving convergence over 1–3 days (To et al., 2007). This asymmetry may result from the properties of the network structure.

### Regular Network

Bernard et al. (2007) used a regular network structure for the SCN, where neuronal nodes were only connected to nodes in local proximity instead of with all other nodes. In this model, the neuronal nodes are damped oscillators rather than self-sustained oscillators and the edges between two nodes do not extend a critical distance. It was found that the size of the networks, i.e., the number of nodes and the number of edges in the SCN network, influences the circadian rhythms as well as the synchronization of the activity patterns of the SCN neurons. If the network is dense (meaning a high number of connections) or the number of neurons is large, the circadian rhythms of the neurons are robust and the synchronization degree is high. If the network is sparse (few links) or the number of neurons is small, the neurons become arrhythmic and synchronization between the activity patterns of the neurons is lost. Therefore, the authors argue that the rhythms of the SCN neurons and the synchronization are co-dependent, and the intracellular clocks cannot be isolated from their intercellular communication (topological neighbors) in the SCN network. Note that all neurons were considered to be homogeneous in nature and all were damped oscillators.

Another example of a regular local network for the SCN is described in Kunz and Achermann (2003). Here the authors used the Van der Pol oscillator model for the single neurons. Also in this locally connected regular network synchronization of the neuronal activity patterns arises, leading to an overall rhythm of the SCN. In this paper the authors state that in a sparsely connected network, noise at the cellular level distorts the SCN rhythms, whereas if the network is dense, the SCN rhythms are robust against this cellular noise. Moreover, the authors observed that a dissociation of one group of neuronal oscillators could arise in constant conditions, which could be a mechanism for the splitting phenomenon (Kunz and Achermann, 2003).

### NW Small-World Network

In Vasalou et al. (2009), it was suggested that the SCN network most probably has small-world properties. The collective behaviors of the SCN neurons are compared between a regular network with only local connections, a NW small-world network with 10% long-range connections, and an all-to-all network. The total number of links in the regular and small-world network only comprise about 5% of the total number of links in the all-to-all network, thus requiring much less energy because the links (signal transmission via action potentials) between two distant neurons consumes high amounts of energy (Attwell and Laughlin, 2001; Vasalou et al., 2009). Despite the lower amount of connections, the small-world network still shows the same functional performance as the all-to-all network, whereas the regular network was not capable of producing proper circadian rhythms. This indicates that the long-range connections play a key role in the circadian function. The loss of long-range connections leads to decreased synchronization between the neurons and reduced circadian function, which may possibly explain circadian dysfunction in aging, as in aging long-range connections are thought to be compromised first. Therefore, the authors suggested that the SCN should be a NW small-world network because this type of network provides an optimal comprise between the density of the network and the control of both circadian amplitude and synchronization (Vasalou et al., 2009).

The NW small-world network was also considered in Webb et al. (2012). In this paper the authors considered heterogeneous neuronal types, with sustained oscillations, weak oscillations and non-oscillating neurons (arrhythmic) as found in Webb et al. (2009). Networks containing more weak oscillators were better able to synchronize than networks with more sustained oscillators. Also if the weak oscillators occupied key positions in the network (having a high number of connections, so being the hubs in the network) the network synchronized better. A higher degree of synchronization also leads to high amplitude oscillations of the whole SCN. The authors concluded that both intrinsic properties of single neurons and the locations of the neurons within the network explain the dynamic emergence of rhythms and synchrony of individual cells.

### Scale-Free Network

**FIGURE 5:**
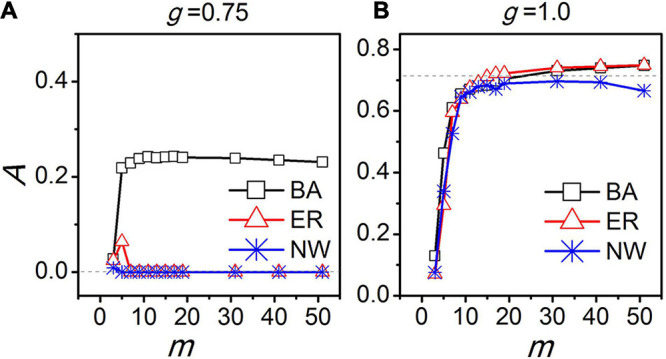
The relationship between the amplitude of the SCN network (*A*) and the average node degree (*m*). “BA”, “ER,” and “NW” represents Barabási-Albert scale free network, Erdos-Renyi random network, and Newman-Watts small-world network, respectively. The dashed lines indicate the amplitude *A* for an all-to-all network. The coupling strength is represented by *g.* The coupling strength *g* = 0.75 corresponds to moderate coupling **(A)** and *g* = 1.0 to strong coupling **(B)**.

In Gu and Yang (2016), the SCN was suggested to be a scale-free network. It was observed that when the coupling between the neurons is weak, the neurons become synchronized in their activity patterns inducing a circadian rhythm in the SCN only in a scale-free network, but not in an all-to-all network, a regular network, or a small-world network. When the cellular coupling is moderate, the SCN amplitude is largest in the scale-free network compared to the other network topologies, although also in these networks a circadian rhythms appears. When the cellular coupling is strong, the SCN rhythm amplitudes are very close between the different types of networks (Figure 5). Note that the total number of links for a scale-free network is evidently smaller than for the all-to-all network. Therefore, we concluded that the network topology but not the number of links determines the robustness of the SCN rhythms, and the density of the links in the SCN network can be sparse (Gu and Yang, 2016).

In Hafner et al. (2012), three types of networks were compared for their effects on the synchronization of activity phases of the neurons and the entrainment properties of the SCN. The network topologies that were investigated were a regular network (which they call a local network), a scale-free network and a random network. Without external light input from the environment (i.e., constant darkness), both the degree of synchronization of the activity phases of the neurons and the SCN amplitude are larger in a scale-free network than in the other two types of networks when the node degree is low. For higher node degree, synchronization and amplitude are similar between these three types of the networks (Hafner et al., 2012). For entrainment to light the authors show that if hub-neurons (neurons with a high outgoing degree) are light-sensitive, the SCN behaves more according to experimental results compared to when randomly picked neurons receive the light information. They show that in the case of the hub neurons being light-sensitive, in accordance with the experiments, the circadian rhythm is more affected by eastbound that westbound-induced jet-lags.

### Communities Divided by the VL and the DM

It is natural to regard the VL and the DM as distinct communities, because of the difference in the sensitivity to light information between both, as well as the difference in internal and external coupling of the neurons in each community. Hafner et al. (2012) investigated a scale-free network divided into an VL and a DM part, each of which was given a different topology. The sensitivity of this network to short pulse-like external perturbations is reduced but for long external perturbations the sensitivity is improved, which is important for the SCN as it should be robust to pulse-like disturbances, while still being flexible enough to entrain to new light-dark regimes.

Vasalou and Henson (2011) also used different topologies for the VL and DM parts of the SCN, where the VL had a small-world topology and the DM a regular topology. The authors explored a threshold for the number of (randomly selected) long-range connections (links) going from the VL to the DM that still achieved synchrony of the activity of the neurons to retain a 24-h rhythm of the SCN in constant darkness and in external light-dark schedules. By contrast, in constant light conditions, the activity patterns of the DM neurons obtain increased synchronization, but the synchronization of the activity patterns of the VL part decreases drastically. Therefore, the authors suggested that a differential light response exists across the two communities.

The differences between VL and DM seem to be important for the function of the SCN, but it can also lead to the dissociation between the VL and the DM if an animal is exposed to a 22 h light-dark T-cycle. Consistent with experimental results from a light-dark T-cycle of 22 h, we observed that an important factor for the dissociation between the VL and the DM is the difference in light-sensitivity between the VL and the DM. We used a mathematical model, which, for simplicity reasons, was coupled with a global mean field coupling, so treating the SCN as an all-to-all network (Gu et al., 2012). Based on the Goodwin model, the dissociation was the result of the VL neurons being light-sensitive and DM neurons were not. Another aspect that affects the dissociation is that the neurons are heterogeneous and not homogeneous. A diversity of the neuronal intrinsic periods and a difference in the amplitudes between the VL neurons and the DM neurons also could introduce the dissociation between the VL and DM (Gu et al., 2012, 2018).

An experimental explanation has not yet been found for the difference in the collective behaviors of the SCN neurons between long and short photoperiod conditions. Based on mathematical models, it was explained that the long-range links between the VL and the DM could play a role in this difference. In Bodenstein et al. (2012), the dense long-range links between the VL and the DM during short winter days leads to better synchronization and higher amplitude than the long summer days where the long-range links are sparse. Additionally, the dense long-range links result in larger entrainment ability (faster phase-shift of the SCN, larger entrainment range and larger area of the phase response curve) during the winter. An alternative explanation for the difference between long and short photoperiod is given by Gu et al. (2016), where the ratio of the number of links going from VL to DM is taken with respect to the DM to VL direction. We assumed that the total number of the links between the VL and the DM was not altered, but the ratio of the number of links from the VL to the DM to this total number is altered. The larger ratio during the short winter days were assumed, and accordingly, a higher synchronization between the VL and the DM is observed, compared to a smaller ratio for long summer days.

### Communities Divided by the Right and the Left SCN

Whereas the SCN regulates the circadian rhythms of behavioral activity, this behavioral activity also feeds back to the SCN network. The feedback is assumed to play a role in the rhythmic behavior of rats. For simplicity, the SCN network is considered to be an all-to-all network which is divided into two communities, i.e., a right SCN and a left SCN, using either a Kuramoto model or a Goodwin model (Indic et al., 2008; Gu et al., 2011). Each SCN neuron is affected in three ways: it is affected by the neurons within the same community, by the neurons from the other community and by the feedback. Accordingly, four key parameters were taken into account, including the external coupling strength between the two communities, the internal coupling strength within each community, feedback strength and the time delay of the feedback. Note that the total strength, i.e., the summation of internal coupling strength, external coupling strength and the feedback strength is assumed to be a constant. Therefore, the number of free parameters is only 2 for the total 3 strength parameters. The combination of these four parameters determines the collective behaviors of the SCN neurons.

The authors found that when the time delay of the feedback is around 12 h, all three phenomena can emerge depending on the values of the three coupling strengths (Indic et al., 2008; Gu et al., 2011). Typically, if both the feedback strength and the internal coupling strength are large, and the external coupling strength is small, the splitting phenomenon emerges, that is, the two communities show about 24 h periods and they are anti-phase. If the feedback strength is large, and both the external and internal coupling strength are moderate or small, the neurons lose the circadian rhythms in that the SCN network becomes arrhythmic. If the feedback strength is small, and either the external or internal coupling strength is large, the circadian rhythm of the SCN network shows robust rhythms because the SCN neurons are synchronized in their phase of activity. Therefore, the feedback with time delay plays a role in the rhythmic behaviors of the SCN network.

However, the feedback from the behavioral activity to the SCN in experiments (multi-unit recordings) was immediate or in a very short time (van Oosterhout et al., 2012). Accordingly, we built a phase model with a feedback which does not include time-delay but a phase angle of the influence of the feedback to the SCN (Gu et al., 2019b). We also observed that the emergence of the rhythmic behaviors of the SCN relies on the vales of the three strengths when the phase angle is close to π.

An alternative explanation for the splitting phenomenon is given in Rohling and Meylahn (2020). They use a noisy Kuramoto model for the neurons operating in two communities, the left and right SCN. It has been shown that for long and short photoperiods the excitation-inhibition (*E*/*I*) balance in the SCN changes, when GABAergic connections between VL and DM in long-day photoperiod shifts from inhibitory to excitatory (Farajnia et al., 2014). The noisy Kuramoto model shows that a change in internal and external communication strengths within and between the communities, which changes the *E*/*I* balance of the SCN, may result in the SCN going from a stable synchronized state to an unstable state (Rohling and Meylahn, 2020). As this state is unstable, the SCN system tries to find a new (semi-)stable state, and in some circumstances this may be the split-state. Thus, a change in the nature of the links can also cause the network to shift towards a new state.

## Discussion and Conclusion

Circadian rhythms are controlled by the SCN. The SCN rhythm originates from the oscillation of the individual neurons, but the function of the SCN depends on the collective behaviors from all of the neurons within the SCN network (Michel and Meijer, 2020). For example, the robust rhythm of the SCN depends on the synchronization between the neurons, the recovery from the jet-lag desynchrony is related to the synchronization of the neuronal communities in the DM and the VL parts of the SCN, and the entrainment of the SCN to external T-cycles relies on the coupling between neurons. Thus, it is important to study the effects of the interaction mechanisms between neurons (i.e., the network topology) on the SCN function.

Here, we reviewed the studies of the SCN network from two aspects. First, the SCN network topology was revealed by the time series analysis of experimental data. It was found that the SCN is composed of two communities which is consistent with anatomical results, and it is shown that the SCN network is characterized by a short average shortest path length (correlation analysis suggests a NW small-world network and transfer entropy analysis suggest a scale free network). Secondly, the effect of SCN network topology on the SCN function was reviewed based on mathematical models.

The SCN network is more complex than the simple network we considered in the present review. We reviewed the relatively static network structure of the SCN on the longer timescale (of a day), which shows in rhythms of gene expression and of daily electrical activity patterns of neurons. The real interactions between the neurons take place on much smaller timescale, milliseconds to seconds. This difference in timescales has been described for GABA coupling where tonic and phasic GABA action works at different time scales (Deoskin et al., 2015). It was found that the network structure of the SCN is not characterized as a small-world network as well as not a scale free network, since the cluster coefficient is small (around 0.1) or the node degrees do not satisfy a power-law distribution (Freeman et al., 2013). Another complicating factor is that there is not only attractive communication in the SCN, but also repulsive communication (Myung et al., 2015), although it should be noted that inhibitory connections between neurons do not necessarily lead to less synchronization in the network. To extend our knowledge on the SCN network, future work should consider a more realistic network structure for the SCN, taking into account the multilayer network for different timescales, including electrical processes and the glial cells, and the attractive links and the repulsive links (Deoskin et al., 2015; Myung et al., 2015; Pauls et al., 2016; Sueviriyapan et al., 2020).

As it is not possibly (yet) to fully infer the network structure of the SCN from experimental results, the causality between network topology and SCN function can also not be established using experimental data. As research awaits advancements of new experimental techniques, mathematical models can be used to compare different network types for the SCN. These mathematical modeling studies found that the network topology is very important for SCN function. Typically, the synchronization degree between neurons and the SCN amplitude are largest in a small-world network or a scale-free network architecture. The results reviewed here shed light on both the SCN network and SCN function, and provides advice for experimental research.

## Author Contributions

All authors listed have made a substantial, direct and intellectual contribution to the work, and approved it for publication.

## Conflict of Interest

The authors declare that the research was conducted in the absence of any commercial or financial relationships that could be construed as a potential conflict of interest.

## Publisher’s Note

All claims expressed in this article are solely those of the authors and do not necessarily represent those of their affiliated organizations, or those of the publisher, the editors and the reviewers. Any product that may be evaluated in this article, or claim that may be made by its manufacturer, is not guaranteed or endorsed by the publisher.
